# An omicron-specific neutralizing antibody test predicts neutralizing activity against XBB 1.5

**DOI:** 10.3389/fimmu.2024.1334250

**Published:** 2024-01-23

**Authors:** Stephen Varvel, Marzena Galdzicka, Stephanie Nystrom, Hong Liu, Ge Chen, Izabela Ragan, Shahrokh Shabahang

**Affiliations:** ^1^ Pearsanta, Inc., Richmond, VA, United States; ^2^ Aditxt, Inc., Mountain View, CA, United States; ^3^ Biomedical Sciences Department, Infectious Disease Research Center, Colorado State University, Fort Collins, CO, United States

**Keywords:** SARS-CoV-2, omicron, neutralizing antibody, flow cytometry, XBB.1.5

## Abstract

**Introduction:**

Understanding the immune status of an individual using neutralizing antibody testing is complicated by the continued evolution of the SARS-CoV-2 virus. Previous work showed that assays developed against the wildtype strain of SARS-CoV-2 were insufficient predictors of neutralization of omicron variants, thus we developed an omicron-specific flow cytometry-based neutralizing antibody test and performed experiments to assess how well it compared to an omicron-specific PRNT assay (gold standard) and whether it could predict neutralizing activity to more recent omicron subvariants such as XBB.1.5.

**Methods:**

Accuracy of a novel flow cytometry-based neutralizing antibody (FC-NAb) assay was determined by comparison with an omicron-specific PRNT assay. A series of samples were evaluated in both the omicron FC-NAb assay and a second test was designed to assess neutralization of XBB.1.5.

**Results:**

Good concordance between the omicron FC-NAb test and the omicron PRNT was demonstrated (AUC = 0.97, p <0.001; sensitivity = 94%, specificity = 100%, PPV = 100%, and NPV = 97%). A strong linear relationship between the omicron FC-NAb and neutralization of XBB1.5 was observed (r = 0.83, p<0.001). Additionally, the omicron FC-NAb test was a very strong predictor of positive XBB1.5 NAb activity (AUC = 0.96, p<0.001; sensitivity = 94%, specificity = 90%, positive predictive value = 90%, and negative predictive values = 94%).

**Discussion:**

Our data suggest that despite continued evolution of the SARS-CoV-2 spike protein, the omicron FC-NAb assay described here is a good predictor of XBB1.5 neutralizing activity, as evidenced by a strong correlation and good predictive performance characteristics.

## Introduction

The global COVID-19 pandemic has led to great interest in new methods for assessing individual’s immune responses to SARS-CoV-2 exposure and vaccination. Of particular interest have been neutralizing antibodies (NAbs), which are the subset of elicited antibodies which attach to SARS-CoV-2’s spike protein receptor binding domain (RBD) and interfere with binding to ACE2 receptors thus inhibiting viral entry into cells. Studies have shown that neutralizing antibodies are good predictors of vaccine efficacy as well as resistance to re-infection (with the same viral strain), and thus serve as a correlate of protection (1–7). Throughout the pandemic, some clinicians have found value in assessing neutralizing antibody levels of patients to help inform vaccine booster and other exposure mitigation strategies. However, mutations in the spike protein have led to SARS-CoV-2 variants capable of substantially evading antibody defenses established in response to vaccination with the original vaccines or exposure to earlier forms of the virus. Thus, understanding the immune status of an individual using neutralizing antibody testing is complicated by the continued evolution of the SARS-CoV-2 virus.

Several methods are used to determine SARS-CoV-2 NAb activity (e.g., 8). The “gold standard” method assessing NAbs is the plaque reduction neutralization test (PRNT), which determines the antibody titer that reduces virus-induced plaques in a cell system. However, the required use of live virus in a Biosafety Level 3 facility and the length of time to perform the assay precludes its utility in a clinical setting. Pseudovirus-based neutralization tests (PBNT) use synthetic viruses that simulate the infection process but do not replicate. These tests can be performed in a Biosafety Level 2 facility, but they remain impractical for most clinical purposes due to time, labor, and cost concerns. In contrast, surrogate virus neutralization tests (sVNT) directly assess the competitive inhibition of SARS-CoV-2 RBD peptides binding to human ACE2 receptors and can be implemented routinely in the laboratory with technologies such as enzyme-linked immunosorbent assays (ELISA), and even as point of care devices with lateral flow immunoassays (e.g., 9, 10). Our group and others have developed flow cytometry-based sVNT systems that allow rapid multiplexed determination of NAb activity against multiple SARS-CoV-2 variant RBDs simultaneously (11, 12). These flow cytometry NAb tests (FC-NAb) are well suited for deployment in a clinical setting and can be relatively easily adapted to include emerging variants of concern. Furthermore, the availability of International reference materials can improve standardization of results between labs.

Since the beginning of 2022, the Omicron variants of the SARS-CoV-2 virus have been the predominant forms of the virus in circulation in the U.S. Studies have shown that higher levels of antibodies formed in response to vaccination or a previous exposure to an earlier form of the virus are required to neutralize Omicron compared to older variants (13–15). Consequently, breakthrough infections with the Omicron variant have been common, even if individuals with adequate wildtype NAb activity (e.g., 16). Importantly, exposure to an Omicron variant of the virus or one of the Omicron-containing bivalent vaccines available in the U.S. since September 2022, will increase neutralizing antibodies specific to the Omicron subvariants (12, 17–19)). We earlier published an analysis of cross-neutralization in our FC-NAb assay to a series of variants of concern and showed that NAb activity against the original (wildtype) strain did not correlate well with NAbs against the Omicron BA.1 and BA.2 subvariants, though they performed well against earlier variants (12).

We subsequently developed an “Omicron-specific” neutralization test that combines both BA.1 (B.1.1.529, the original omicron variant) and BA.2 RBD peptide coated beads and have introduced this test into clinical practice at relatively low cost and rapid turnaround time (less than 4 hours). Since that time, Omicron has continued to evolve. XBB1.5, another Omicron subvariant, emerged in the United States at the end of December 2022 and quickly became the dominant SARS-CoV-2 variant. XBB1.5 demonstrated enhanced escape from antibodies elicited by earlier forms of SARS-CoV-2 or the vaccines and in some models showed significant escape from antibodies produced even by infection with earlier Omicron subvariants (e.g. 20–22). Updated vaccines from Pfizer, Moderna, and Novovax are formulated against the XBB.1.5 subvariant. Thus, we elected to perform additional experiments to determine the correlation between neutralizing antibody activity against the earlier Omicron versus the XBB1.5 subvariants.

## Materials and methods

### Flow cytometry

The Test for Neutralizing Antibodies to SARS-CoV-2 used in this study is a multiplex flow cytometry-based competitive inhibition neutralizing antibody assay (FC-NAb) designed to measure the ability of antibodies in a patient sample to inhibit the binding of labeled ACE2 receptors to SARS-CoV-2 RBD peptides. Each sample was incubated with a SARS-CoV-2 variant bead array and Phycoerythrin(PE)-labeled ACE2 stain buffer for 1 hour (All reagents made in-house). After 2 washes with wash buffer, the bead array was acquired on a BD FACSLyric™ Flow Cytometer (acquiring 500 beads per RBD antigen bead population) and median florescence intensity (MFI) was measured; neutralizing Ab (NAb) activity was calculated by the following formula: NAb (%) = [Negative control(MFI) – Sample (MFI)]/Negative control (MFI) X 100%]. For the primary bead array, percent inhibitions of B.1.1.529 (BA.1) and BA.2 were averaged for an “Omicron-specific” result and reported separately from neutralization of the wildtype RBD.

The flow cytometry instrumentation used in this study was the BD FACSLyric [BD Biosciences; 3-laser (blue- 488 nm, red- 640 nm, violet- 405 nm); 10 colors]. NAb detection Assay/Tube Settings Setup was manually created using negative control samples to set PMT voltages. The three bead populations corresponding to RBD WT and Omicron BA1 an BA2 were gated separately based on their different fluorescence intensities in UV and violet channels; Phycoerythrin was used as fluorescent reporter. The lack of interference associated with testing these antigens at the same time was demonstrated by comparing assessed NAb activity when SARS-CoV-2 RBD wildtype, BA.1, and BA.2 were tested individually (in single bead assays) with results obtained from the multiplexed bead array (see **Supplementary Table 1**). Compensation was set using BD CS&T beads (BD Biosciences) and BD FC beads (BD Biosciences), automatically calculated by BD FACSuite software (BD Biosciences) and applied to the NAb detection assay during daily QC and Assay/Tube Settings Setup to achieve consistent assay performance from one day to another. Intra-assay and Inter-assay precision during test validation was <20% (see **Supplementary Table 2**).

### Preparation of RBD-coated microparticles

SARS-CoV-2 RBD proteins were conjugated onto polystyrene microparticles with different fluorescence IDs (Spherotech, Lake Forest, IL 60045) by the Two-Step EDC (Pierce biotechnology, Rockford, IL 61105) conjugation protocol. The primary bead array consisted of SARS-CoV-2 wildtype (Cat# 19CoV-S120, ExonBio, San Diego, CA 92121), B.1.1.529 (Cat# SPD-C522e, AcroBiosystems, Newark, DE, 19711), and BA.2 (Cat# SPD-C522g, AcroBiosystems, Newark, DE, 19711) RBD antigen coated beads (12). A second bead set containing RBD antigen specific to wildtype and XBB1.5 (Cat. #SPD-C5242, AcroBiosystems, Newark, DE, 19711) was developed for comparison studies. For analyses of performance characteristics, XBB1.5 NAb activity was considered positive if there was >33% inhibition (based on the earlier work associating % neutralization with reduced COVID-19 incidence; 4).

### Plaque reduction neutralization Assay (PRNT)

Production of neutralizing antibodies was determined by a plaque reduction neutralization test. Briefly, serum was first heat-inactivated for 30 minutes at 56°C in a water bath. Serum samples were diluted two-fold in dilution media containing DMEM +1%FBS + antibiotics starting at a 1:5 dilution on a 96-well plate. An equal volume of SARS-CoV-2 virus (Isolate hCoV-19/USA/MD-HP20874/2021 Lineage B.1.1.529; Omicron Variant, BEI Resources NR-56461) equivalent to 100 plaque forming units per well was added to the serum dilutions, and the sample-virus mixture was gently mixed. Plates were incubated with occasional rocking for 1 hour at 37°C. Following incubation, serum-virus mixtures were plated on confluent Vero E6 cells. The plates were incubated with occasional rocking for 1 hour and then overlaid with 0.5% agarose in media with 7.5% bicarbonate and incubated for 1 day at 37°C, 5% CO2. A second overlay with Neutral Red stain was added and plates were incubated for an additional 48-72 hours to allow for plaque formation and visualization. Antibody titers were recorded as the highest dilution in which >50% and >90% of virus was neutralized. Antibody titers detected at 1:10 or higher dilution were considered positive.

### Determination of the standard curve

A standard curve was determined using WHO’s 1st International Standard for antibodies to SARS-CoV-2 variants of concern (NIBSC code: 21/338), which included a standardized Omicron NAb value in IU/mL. Serial dilutions of the standard were assessed with the Omicron FC-NAb assay. Each dilution was run in 6 replicates on 3 instruments (18x total).

### Study subjects

For comparisons between Omicron (BA.1/BA.2) and XBB.1.5 NAb activity (FC-NAb), samples (N=176) were selected from a set of study samples obtained as part of a larger prospective study evaluating SARS-CoV-2 immune responses (NCT05379478, WCG IRB - #20202768). Written informed consent was provided. Specific samples collected between Sep 2021 – May 2023 were selected to provide a range of NAb values, a good balance of age and gender, and to enrich the set with subjects who had COVID-19 during 2023 (when XBB1.5 was prevalent) or had received one of the bivalent COVID-19 vaccine boosters. Characteristics of subjects included in these comparisons are shown in **Table 1**. An additional set of de-identified routine clinical samples (N=91) chosen based on their Omicron NAb activity and representing the full range of possible values was selected for comparison between the FC-NAb and PRNT assays. Samples were stored at -80^○^C until testing.

**Table 1:** Characteristics of subjects whose samples were included in the comparison between omicron (BA.1/BA.2) and XBB.1.5 NAb activity in the FC-NAb assay.

Characteristic	Group 1 No vaccine, no known infection	Group 2 No vaccine, Previous Infection	Group 3 Vaccine, no known infection	Group 4 Vaccine, Previous Infection	Total
**N** **(Subjects/Samples)**	10/11	31/32	26/53	41/80	108/176
**Age** **(mean/std)**	44.4 (15.4)	48.4 (12.7)	53.9 (14.0)	52.9 (13.6)	50.8 (13.8)
**Sex** **(% Female)**	60.0%	48.4%	63.0%	48.8%	54.6%
**Ethnicity** **(% White)**	90.1%	96.7%	78.3%	87.5%	88.9%
**BMI** **(mean/std)**	24.1 (5.6)	26.2 (5.0)	28.5 (5.4)	29.8 (6.5)	27.8 (6.0)
**Months since last vaccine** **(mean/std)**	N/A	N/A	6.5 (6.6)	11.4 (7.1)	N/A
**% bivalent vaccines**	N/A	N/A	37.3%	13.5%	N/A
**Months since last infection** **(mean/std)**	N/A	7.2 (5.8)	N/A	6.8 (7.7)	N/A
**% Omicron infection**	N/A	25.9%	N/A	78.8%	N/A

Demographic information is provided per subject, information about the timing of infection and/or vaccinations are provided per sample.

### Statistical analysis

Analysis of Variance (ANOVA), ROC analysis, determination of performance characteristics and linear regression were performed in MedCalc (v19.6.4, MedCalc Software Ltd, Belgium), while analysis of variance was performed in Prism (v9; GraphPad Software, San Diego, CA, USA).

## Results

**Figure 1:**
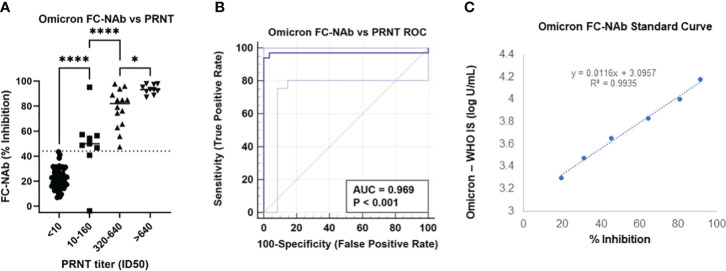
Comparison of the Omicron FC-NAb assay with an Omicron PRNT reference method and determination of a standard curve compared to an omicron-specific WHO NAb international standard. **(A)** Omicron NAb activity (% inhibition) determined with the FC-NAb assay is shown by PRNT NAb titer (ID_50_) category. The center line of each category depicts the median FC-NAb % inhibition. The dotted line (at 44% inhibition) represents the clinical cut-off. Asterisks denote significant group differences determined by Tukey’s multiple comparison tests, * p<0.05, **** p<0.0001. **(B)** Receiver Operating Characteristic (ROC) curve generated from the same data shows the Omicron FC-NAb asay predicting positive Omicron PRNT results, AUC = 0.97, p<0.001. **(C)** A standard curve relating dilutions of the NIBSC omicron reference material concentrations to FC-NAb % inhibition.

The FC-NAbs assay was validated in our a CLIA-certified, CAP-accredited laboratory, and used for this study. The accuracy of the FC-NAb assay was determined by comparing our Omicron NAb results with neutralizing titers obtained with the “gold standard” PRNT. As shown in **Figure 1A**, FC-NAb % Inhibition increased significantly as PRNT ID_50_ titers increased (F[3,87] = 5.2, p<0.0001). Tukey’s multiple comparisons test showed that % Inhibition increased significantly at each successive ID50 category compared to the one before it. Performance characteristics showed good concordance between the Omicron FC-NAb test and the Omicron PRNT, as shown in **Figure 1B** (Receiver operating characteristic [ROC] analysis, AUC = 0.97, p <0.001). When 44% Inhibition was used as the cut-off, the Omicron FC-NAb test was able to identify positive PRNT samples (any detectible Omicron neutralization with an ID_50_ >10) with a sensitivity = 94%, specificity = 100%, PPV = 100%, and NPV = 97%.

The standard curve was established using WHO’s 1st International Standard for antibodies to SARS-CoV-2 variants of concern (NIBSC code: 21/338) that included a standardized Omicron NAb value in IU/mL. The conversion of % inhibition to IU/mL was determined as log U/mL = [0.0116 x % Inhibition] + 3.0957), R^2 =^ 0.99, **Figure 1B**.

**Figure 2:**
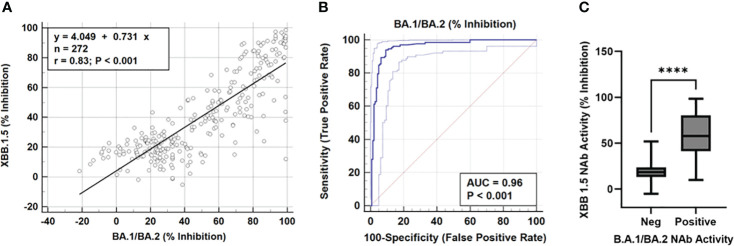
**(A)** Scatter diagram and regression line showing NAb activity (% inhibition) of omicron (BA.1/BA.2) compared to XBB.1.5 in the FC-NAb assay, r = 0.83, p <0.001. **(B)** Receiver operating characteristic (ROC) curve showing the omicron FC-NAb as a predictor of XBB1.5 positivity, AUC = 0.96, p<0.001. With a cutpoint of 44% inhibition, omicron NAbs predicted positive XBB neutralizing activity with a sensitivity of 94%, specificity of 90%, positive predictive value of 90%, and negative predictive values of 94% **(C)** XBB.1.5 NAb activity in subjects with negative or positive omicron FCNA neutralizing activity, **** indicates p <0.0001.

Comparisons between the Omicron FC-Nab test and XBB1.5 neutralizing activity are shown in **Figure 2**. A strong linear relationship between the Omicron FC-NAb and neutralization of XBB1.5 was observed (r = 0.83, p<0.001, **Figure 2A**). Receiver operator characteristic (ROC) analysis determined that Omicron Nabs were a very strong predictor of positive XBB1.5 NAb activity (AUC = 0.96, p<0.001) with an optimal Omicron NAb cut point at 44% inhibition. At this cutpoint, Omicron NAbs performed with a sensitivity of 94%, specificity of 90%, positive predictive value of 90%, and negative predictive values of 94% (**Figure 2A**). When samples were categorized based on Omicron NAb values, significant increases in XBB1.5 NAbs were seen in the positive group (>44% Inhibition), but not in the weak positive group (33-44% Inhibition); (F[2,177]=39.1, p<0.0001; **Figure 1**).

**Figure 3:**
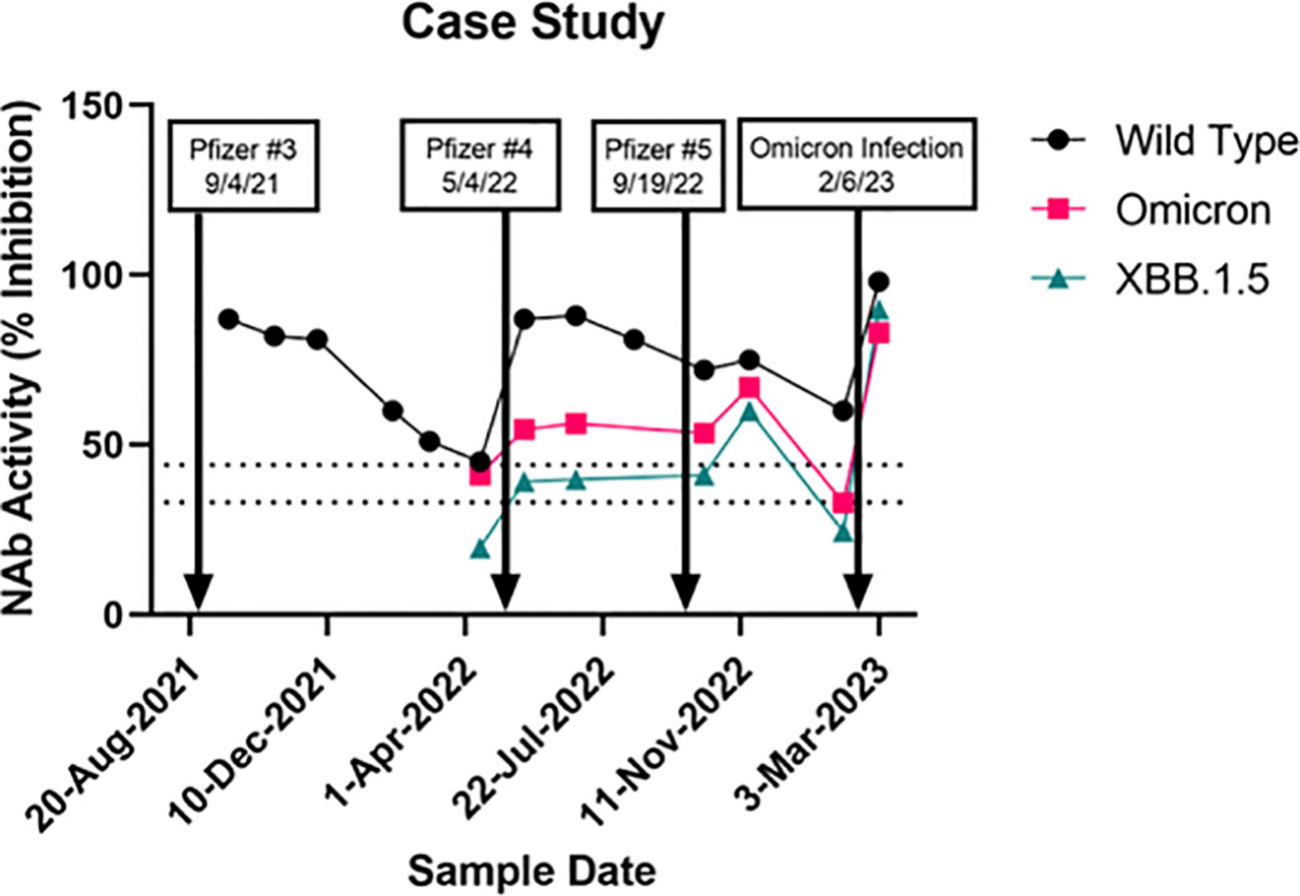
A case study of a subject followed for one and a half years showing effects of a series of vaccine boosters (the 9/19/22 shot was a Pfizer bivalent booster) and an omicron infection. Neutralizing antibody activity (% inhibition) against wildtype, omicron (BA.1/BA.2), and XBB.1.5.

NAb activity against wildtype, Omicron, and XBB.1.5 in a study subject with a longitudinal set of samples that spanned several vaccine boosters, and a subsequent Omicron infection is shown in **Figure 3**. The first sample analyzed shortly after a Pfizer booster (after an initial 2-shot regimen earlier in the year) showed near maximal wildtype NAb activity which waned steadily over the next 7 months. A second booster administered May 4, 2022, elicited a strong wildtype response with much smaller increases in Omicron and XBB1.5 NAbs. Four months later a third booster, which contained the bivalent wildtype + Omicron formulation, was administered. Little to no further increase in wildtype NAbs were observed, though both Omicron and XBB.1.5 NAbs showed additional increases in NAbs. A sample obtained just 3 months later showed these increases were short-lived, as both Omicron and XBB.1.5 NAbs had dropped below 44% inhibition. A breakthrough COVID-19 infection began just a week later, which elicited strong NAb responses to wildtype, Omicron, and XBB.1.5. While the variant involved in this breakthrough infection was not determined, it is likely to have been XBB.1.5 according to the variant prevalence data provided by the CDC’s COVID Data Tracker (23).

## Discussion

Here we report data from a FC-NAb assay with utility in assessing Omicron-specific NAb responses to vaccination or infection and in estimating current levels of protection against future infection. NAb results from the FC-NAb test were concordant with the PRNT gold standard, and results were in alignment with the WHO standard into standardized units (IU/mL). Importantly, our Omicron NAb results showed good correlation with XBB.1.5-specific NAb activity.

In the early days of the pandemic, lack of standardization among SARS-CoV-2 antibody assays was a challenge to the successful implementation of antibody testing strategies for vaccine development and clinical monitoring of immune responses to infection and vaccination (24). In Dec 2020, the WHO Expert Committee on Biological Standardization (ECBS) established the 1st WHO International Standard (IS) for anti-SARS-CoV-2 immunoglobulin (NIBSC code 20/136) (25). Due to depletion of the original standard material and the emergence of SARS-CoV-2 variants, two candidate preparations were evaluated as a potential new IS. One of these (NIBSC code 21/340) was determined to be as suitable as the 2^nd^ WHO IS due to its traceability to the original IS - though it was not deemed appropriate for Omicron and its subvariants. Instead, the other candidate (NIBSC code 21/338, originally identified as a “working Standard for anti-SARS-CoV-2 antibodies”) was classified as the “1^st^ WHO IS for antibodies to SARS-CoV-2 variants of concern” and assigned a unitage of 17,000 IU/mL for neutralizing antibody activity against Omicron sublineages and other variants of concern emerging after June 2022 (26). The availability of this international standard for Omicron NAbs has been an important step in the development of quantitative omicron-based NAb assays enabling results to be compared across laboratories and platforms.

Interest in SARs-CoV-2 NAb assays has led to a large number of commercially available sVNT kits and laboratory services. For example, a recent comparison of three different commercially available sVNT kits evaluated two ELISA based tests (from GenScript and Dynamiker) and a bead-based chemiluminescence assay (Mindray Ntab) (27). All three tests demonstrated good performance against the PRNT. However, these tests measured neutralization of the original SARS-CoV-2 virus, and were assessed with samples obtained early in the pandemic or following vaccination with the original formulations of mRNA vaccines. Thus these tests are not appropriate for predicting levels of protection against variants known to evade those antibodies. A notable exception is GenScript’s sVNT ELISA which can target BA.1 and BA.2 RBD, available as a RUO product. Our omicron test performs very similarly to this GenScript assay (PPA = 97%, NPA = 95%,data not shown), and is more readily scalable through automation. To our knowledge, there is no other omicron-based sVNT laboratory service currently on the market.

**Figure 4:**
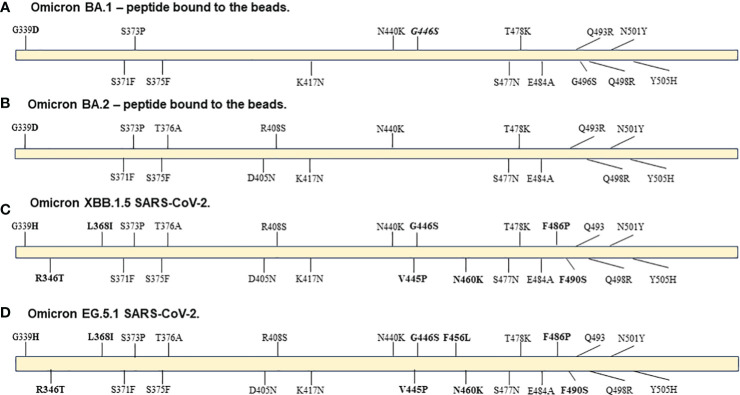
Diagrams of mutations in Spike RBD region AA: Arg 319 - Lys 537 in: **(A)** peptide from ACRO Biosystems Omicron SARS-CoV-2 variant BA.1, **(B)** peptide from ACRO Biosystems Omicron SARS-CoV-2 variant BA.2, **(C)** Omicron variant XBB 1.5 GenBank: WMR94958 and **(D)** Omicron variant EG 5.1 GenBank: WND69405.1. Bolded are mutations in XBB.1.5 and EG.5.1 that are not included in either the BA.1 or BA.2 peptide. The Q493 label means that the original amino acid is present in the XBB.1.5 and EG.5.1 variants even though the BA.1 and BA.2 ACRO peptides have the mutation. The G446S mutation (italicized label) was in the BA.1 peptide and is present in XBB.1.5 and EG.5.1 viral sequences but it is not present in the BA.2 peptide.

Accumulation of multiple mutations in the SARS-CoV-2 spike protein, and particularly the receptor binding domain (RBD), likely drive observed antibody escape and underly the large number of breakthrough infections in vaccinated and/or convalesced individuals observed as the Omicron variants became prevalent at the beginning of 2022. While subvariants of Omicron such as XBB 1.5 continue to accumulate additional mutations that lead them even further from the wildtype strain, the relative number of mutations compared to early Omicron subvariants is substantially less than those between the first Omicron variant and the wildtype version of SARS-CoV-2. As shown in **Figure 4**, there were 15 and 16 mutations in comparison to the wildtype sequence in the B.1.1.529 and BA.2 RBD peptide (AA: Arg 319 - Lys 537) from ACRO Biosystems, respectively, bound to the beads used in our FC-NAb assay. This number of mutations caused the correlation of NAb activities of wild type and BA.2 in our study to be relatively low (r=0.76 p<0.001; data not shown) as shown previously (12). Comparing the sequence of the BA.2 peptide to the XBB 1.5 variant, we found only 8 differences in this region, and consequently the correlation of BA.2 with XBB 1.5 activity is higher (r = 0.83, p <0.001). Even more recent circulating variants such as EG.5 and HV.1 have emerged from the XBB lineage. While not every mutation is likely to have an equal effect on inhibiting antibody binding, it could be expected that EG5.1, in which we only identified 1 additional difference compared to XBB.1.5, would have a similar neutralization profile as XBB 1.5.

Though XBB.1.5 prevalence has receded over the summer, the FDA has recently approved a new set of XBB.1.5 based monovalent vaccine boosters based in part on a belief that they will continue to provide protective immunity against new variants that may emerge during the fall and winter (e.g. EG 5.1). While reports from human studies are forthcoming, published studies in mice suggest monovalent XBB.1.5 vaccination elicits broad neutralizing activity against a range of XBB subvariants, including EG.5.1 (e.g., 28, 29). The Omicron NAb test described here can be a useful tool in determining pre- and post-booster immune responses and the degree in which they neutralize new variants. Huang and coworkers (28) attributed, at least in part, the decreased effectiveness of COVID and flu vaccines to immune imprinting, where pre-existing immunity to earlier strains of a virus may confound immune responses to antigenically distinct future variants. In this study, we have presented a test that can serve as a tool to monitor NAb levels and when those NAbs become less effective because of substantial changes in a newly emerged variant. Furthermore, the assay can be modified by adding new beads to measure NAb activity against a new and distinct variant.

The evolution of SARS-CoV-2 requires continued monitoring when evaluating the degree of protection afforded by neutralizing antibodies established against earlier forms of the virus. For example, Faraone et al. (30) recently reported that EG.5.1 and XBB.2.3 showed similar antibody escape in bivalent (BA.4/5) vaccinated patients and convalescent patients infected during the BA4/5 wave compared to other XBB-lineage subvariants. Interestingly, they were not effectively neutralized with sera from a small number of patients infected during the XBB.1.5 wave (though neither was XBB.1.5). While concentrations of NAbs were not assessed in this study, it is worth noting that the 3 (of 8) patients tested that did show significant neutralization against EG.5.1 had received at least 3 vaccine doses, while the rest had received either only two or no vaccine doses (Farone et al., 2023).

A strength of the platform described here is the relative ease with which it can be modified to include antigen of new variants. However, by carefully comparing the degree of cross-neutralization among variants and subvariants to which clinical tests have already been developed, laboratorians can direct efforts to develop new tests only when existing tests no longer provide significant clinical information. This monitoring system will create a more efficient method for keeping track of variants and to determine when existing tests outlive their utility requiring modification.

Our data suggest that despite continued mutation of the SARS-CoV-2 spike protein, the Omicron FC-NAb assay described here is a useful predictor of XBB.1.5 neutralizing activity, as evidenced by a strong correlation and strong predictive performance characteristics. Good concordance with the gold standard PRNT and the ability to report results in units aligned with an international standard are important features. Thus, this test may be useful for clinicians assessing individual responses to new XBB.1.5-based monovalent vaccines and for estimating a patient’s degree of protection against XBB-lineage Omicron subvariants. As new variants continue to evolve, the correlation of the FC-NAb test in its current form will be evaluated and modifications will be made as needed.

## Data availability statement

The raw data supporting the conclusions of this article will be made available by the authors, without undue reservation.

## Ethics statement

The studies involving humans were approved by WCG IRB - OHRP/FDA IRB registration number (IRB00000533). The studies were conducted in accordance with the local legislation and institutional requirements. The participants provided their written informed consent to participate in this study.

## Author contributions

SV: Formal analysis, Visualization, Writing – original draft. MG: Conceptualization, Supervision, Visualization, Writing – review & editing. SN: Investigation, Writing – review & editing. HL: Investigation, Writing – review & editing. GC: Conceptualization, Supervision, Writing – review & editing. IR: Supervision, Writing – review & editing. SS: Conceptualization, Writing – review & editing.
