# Supplementary material for: An omicron-specific neutralizing antibody test predicts neutralizing activity against XBB 1.5
Source: Front Immunol. 2024 Jan 23;15:1334250. doi: 10.3389/fimmu.2024.1334250 (PMC10845052; doi:10.3389/fimmu.2024.1334250)
Supplement: Supplementary file 1 [file Table_1.docx]

**Supplemental Table 1.** Comparison of NAb activity (% Inhibition) for SARS-CoV-2 RBD wildtype, BA.1, and BA.2 peptides in the FC-NAb assay when evaluated individually or as part of the multiplexed bead array.

| **Sample ID** | **RBD WT (% Inhibition)** | | **RBD BA.1 (% Inhibition)** | | **RBD BA.2 (% Inhibition)** | |
| --- | --- | --- | --- | --- | --- | --- |
|  | Single bead | Bead array | Single bead | Bead array | Single bead | Bead array |
| **1** | 72 | 67 | 0 | 0 | 0 | 0 |
| **2** | 0 | 1 | 0 | 0 | 0 | 0 |
| **3** | 0 | 0 | 0 | 0 | 0 | 0 |
| **4** | 42 | 45 | 0 | 0 | 0 | 0 |
| **5** | 94 | 93 | 0 | 0 | 0 | 0 |
| **6** | 60 | 58 | 0 | 0 | 0 | 0 |
| **7** | 98 | 98 | 71 | 75 | 79 | 81 |
| **8** | 51 | 54 | 0 | 0 | 26 | 28 |
| **9** | 98 | 98 | 86 | 88 | 87 | 88 |
| **10** | 97 | 97 | 64 | 69 | 68 | 70 |
