# Supplementary material for: An omicron-specific neutralizing antibody test predicts neutralizing activity against XBB 1.5
Source: Front Immunol. 2024 Jan 23;15:1334250. doi: 10.3389/fimmu.2024.1334250 (PMC10845052; doi:10.3389/fimmu.2024.1334250)
Supplement: Supplementary file 2 [file Table_2.docx]

**Supplemental Table 2.** Intra-assay and Inter-assay precision of the omicron FC-NAb test on three separate BD FACSLyric instruments. Intra-assay precision was conducted on high positive and low positive controls run in triplicate, and 20 patient samples for each level run in duplicate. Inter-assay precision was conducted on high positive and low positive controls run in triplicate and 20 patient samples for each level run in duplicate twice a day for 3 days.

| **Instrument ID** | **Intra-assay Precision** | | | | **Inter-assay Precision** | | | |  |
| --- | --- | --- | --- | --- | --- | --- | --- | --- | --- |
|  | **Control samples** | **CV (%)** | **Patient samples** | **CV (%)** | **Control samples** | **CV (%)** | **Patient samples** | **CV (%)** |  |
| L1 | High Positive | 3 | High Positive | 0.88 | High Positive | 7.68 | High Positive | 5.63 |  |
|  |  |  |  |  |  |  |  |  |  |
|  |  |  | Medium Positive | 8.12 |  |  | Medium Positive | 6.59 |  |
|  | Low Positive | 3.5 |  |  | Low Positive | 9.00 |  |  |  |
|  |  |  | Low Positive | 16.69 |  |  | Low Positive | 11.38 |  |
|  |  |  |  |  |  |  |  |  |  |
| L2 | High Positive | 6.1 | High Positive | 0.81 | High Positive | 7.16 | High Positive | 3.01 |  |
|  |  |  |  |  |  |  |  |  |  |
|  |  |  | Medium Positive | 8.25 |  |  | Medium Positive | 10.86 |  |
|  | Low Positive | 12.63 |  |  | Low Positive | 11.25 |  |  |  |
|  |  |  | Low Positive | 15.97 |  |  | Low Positive | 7.07 |  |
|  |  |  |  |  |  |  |  |  |  |
| L3 | High Positive | 3.38 | High Positive | 0.85 | High Positive | 3.90 | High Positive | 3.30 |  |
|  |  |  |  |  |  |  |  |  |  |
|  |  |  | Medium Positive | 5.56 |  |  | Medium Positive | 8.90 |  |
|  | Low Positive | 2.43 |  |  | Low Positive | 6.92 |  |  |  |
|  |  |  | Low Positive | 16.50 |  |  | Low Positive | 11.60 |  |
|  |  |  |  |  |  |  |  |  |  |
